# Red Tape and Community Workers’ Proactive Behavior During COVID-19: Applying the Job Demands–Resources Model

**DOI:** 10.3389/fpsyg.2022.871025

**Published:** 2022-06-30

**Authors:** Wei Hu, Shengjie Zhang, Songbo Liu

**Affiliations:** ^1^School of Public Administration and Policy, Renmin University of China, Beijing, China; ^2^School of Labor and Human Resources, Renmin University of China, Beijing, China

**Keywords:** proactive behavior, red tape, job demands–resources model, lack of goal progress, public service motivation

## Abstract

Since the outbreak of COVID-19, community workers’ proactive behavior has played a noteworthy role in the crisis response. Previous research has not highlighted this group and their proactive behavior. To address this important gap, drawing upon the job demands–resources (JD–R) model, this study explores how red tape affects proactive behavior and investigates the mediating role of lack of goal progress (LOGP) and the moderating role of public service motivation (PSM) in this relationship. Based on a two-wave survey with a sample of 656 community workers in China, we found a negative relationship between red tape and proactive behavior. Moreover, this study showed that LOGP mediated this relationship. Contrary to our hypothesis, PSM did not moderate the relationship between LOGP and proactive behavior. These findings have important theoretical and practical implications and can better inform community work during COVID-19.

## Introduction

In the fight against the COVID-19 pandemic, community workers have made a unique contribution to restricting the spread of the pandemic (Paul et al., 2020; Si et al., 2021). In mainland China, their significant efforts have included communicating pandemic prevention knowledge, offering psychological counseling, disinfecting public areas and public facilities, providing services for the elderly and the disabled, and investigating potential risks (Miao et al., 2021). With an average of six workers per 350 people in the community, under the strict pandemic prevention and control policies, community workers have been expected to respond actively to changing conditions and challenges in a complex environment; therefore, taking initiative and engaging in proactive behavior are of great importance (Chen et al., 2021).

Proactive behavior refers to employees’ self-initiated, future-focused, and change-oriented behavior in the workplace (Parker et al., 2006; Parker and Collins, 2010); further, it contributes to the effectiveness of organizational responses amid changing conditions (Crant, 2000; Grant et al., 2009). Proactive behavior also has positive effects on employees; for example, it is associated with positive emotions (Fritz and Sonnentag, 2009), increased innovative behavior (Suseno et al., 2020), and reduced behavioral disorders (Utley et al., 2002). A majority of the existing literature has focused on the influencing factors of proactive behavior, with researchers broadly classifying them into individual differences and organizational contexts. From an individual perspective, sense of self-efficacy and responsibility, degree of expert power, perceived role width (Parker et al., 2019), strong prosocial value (Grant et al., 2009), and psychological flexibility (Kuo et al., 2018) have a positive impact on proactive behavior. In contrast, at the organizational level, investment in employee development (McAllister et al., 2007), high autonomy and transformational leadership (Den Hartog and Belschak, 2012), innovative environment (Segarra-Ciprés et al., 2019), and distributed leadership (Xu et al., 2021) have a positive influence on proactive behavior.

Although several scholars and practitioners have placed emphasis on the influencing factors of proactive behavior, these studies tend to focus on corporations and ignore public organizations, where unnecessary and overelaborate formalities are more prominent and negatively affect employees’ active behaviors and performance (Brewer and Walker, 2013; Kaufmann et al., 2018). Red tape—the complex, time-consuming formalities employees must deal with—has not been similarly highlighted. To fill this gap, this study applies the job demands–resources (JD–R) model (Bakker and Demerouti, 2007) to assess the relationship between red tape and proactive behavior. We consider red tape as the job demand and examine the mediating effect of lack of goal progress (LOGP) associated with this relationship. Scholars believe that employees with high public service motivation (PSM) are likely to engage in spontaneous, innovative behaviors on behalf of the organization (Perry and Wise, 1990). Therefore, in line with other scholars, we also assume PSM as a type of personal resource (Bakker and Demerouti, 2017) and examine whether the relationship between LOGP and proactive behavior is moderated by PSM.

Our work makes the following contributions. First and foremost, we contribute to literature on the causes of proactive behavior. As mentioned earlier, previous studies have primarily focused on private organizations. We introduce red tape, a highly negative factor in public organizations, into the discourse and analyze its impact on community workers’ proactive behavior through the mediating role of LOGP. Second, drawing on literature on public administration, we extend the JD–R model by introducing red tape as a job demand and PSM as a personal resource, thus examining the practical implications of the model (Kernaghan, 2011; Lavigna, 2015; Perry and Vandenabeele, 2015). Third, our study enriches literature regarding red tape. Although the phenomenon of red tape in public organizations has been examined in numerous empirical studies (Pandey and Scott, 2002; Bozeman and Feeney, 2011), these studies have mainly considered job satisfaction, organizational sense of belonging, democratic rights, and organizational performance rather than investigating red tape’s influence on proactive work behavior. Finally, we expand the research on PSM by finding that PSM doesn’t moderate the relationship between LOGP and proactive behavior. Our study broadens both theoretical and empirical knowledge by uncovering the possible reasons for this.

## Theoretical Framework and Hypotheses

### The Job Demands–Resources Model

The JD–R model (Demerouti et al., 2001) classifies working environment elements into job demands and job resources, as factors that lead to positive or negative outcomes. Job demands are physical, psychological, social, or organizational aspects of the job that can cause physical or psychological exhaustion; therefore, they are associated with certain physiological or psychological costs. Correspondingly, job resources are material, psychological, social, or organizational resources provided by the organization to stimulate personal ability, learning, work enthusiasm, and work involvement; they can alleviate the problem of job burnout caused by excessive job demands (Borst et al., 2019). The JD–R model is widely accepted in academia. While it focuses on job requirements and job resources as the two essential job characteristics that affect job burnout, it allows for other factors to influence the relationship.

Considering the assumption of most psychological research that human behavior is the result of an interaction between personal and environmental factors, it is necessary to integrate personal resources into the JD–R model (Schaufeli and Taris, 2014; Bakker and Demerouti, 2017). Bakker and Demerouti (2017) defined personal resources as the beliefs people hold regarding how much control they have over their environment, and emphasized that they play a similar role to job resources. By introducing personal resources into the JD–R model, this theory has been enriched.

Moreover, when scholars first proposed the JD–R model, they did “not take the specific circumstances of certain occupations and contexts into account” (Borst et al., 2019, p. 2). Since most existing research focuses on private organizations, scholars have called for more emphasis on public administration (Kernaghan, 2011; Lavigna, 2015; Perry and Vandenabeele, 2015). Therefore, our study introduces unique but popular public concepts of red tape and PSM into the JD–R model, as a job demand and a personal resource, respectively, to explore how they influence community workers’ proactive behavior.

### Red Tape and Proactive Behavior

Bozeman (1993) provided the first clear definition of red tape, delineating it as the rules, regulations, and procedures that are in force and need to be obeyed, but which do not contribute to the fulfillment of their intended purpose. Since then, scholars have added to this definition, noting that red tape is the distortion or alienation of the function of effective rules and the presence of redundant rules and procedures in organizations. Red tape often includes highly formalized, restrictive, excessive, or meaningless paperwork, in addition to inefficient rules, procedures, and regulations (Hong, 2020). It damages the interests of stakeholders and hinders the realization of legitimate organizational goals, resulting in a series of negative effects (Kaufmann et al., 2019; van Eijk et al., 2019; Blom, 2020). Therefore, red tape is a job stressor that inhibits an individual’s proactive behavior (Crawford et al., 2010; Quratulain and Khan, 2015).

Based on the JD–R model, job demands are unique predictors of (dis)engagement (Bakker and Demerouti, 2017), which in turn predict future proactive behavior (Mohsin, 2019). In the face of the complex and challenging circumstances of the pandemic, community workers need to be more self-motivated and enhance active behaviors such as providing timely help to the community, identifying potential problems in real time, learning from experiences, and voicing better solutions. These kinds of proactive behavior need time, energy, and courage to respond to the constantly changing conditions. However, most community workers in China encountered red tape, including filling out forms, excessive meetings, and endless inspection by superiors (Li, 2020). One community worker said, “When the pandemic was severe, we had to fill in more than a dozen forms a day, which were issued by different departments with basically the same content but slightly different formats and styles.” (Chu and Zhang, 2020, p. 55). From the perspective of resource consumption, red tape consumes precious time and energy, dampening community workers’ enthusiasm. When these workers are faced with mandatory and unexpected additional burdens, they are bound to consume a large number of their resources, making them unable to devote sufficient resources to other activities (Hu and Tang, 2021). Even if they come up with new ideas, it is hard to turn them into concrete initiatives without follow-up resources (DeHart-Davis and Pandey, 2005). Therefore, this study proposes the first hypothesis.

*Hypothesis 1: Red tape has a negative impact on community workers’ proactive behavior.*

### The Mediating Role of Perceived Lack of Goal Progress

Personal goals are the results that a person expects to achieve; they guide decisions and the evaluation of work results (Robbins and Coulter, 2007), and ultimately, affect one’s well-being (Wiese and Freund, 2005). Individuals must deal with different tasks and assume different responsibilities every day, which involves accomplishing multiple goals that may sometimes conflict with each other; they need to allocate time and energy among different tasks. Allocating attention and resources to different tasks requires constant self-control and consideration of how to prioritize the use of resources to promote the realization of work goals (Koopman et al., 2016; Gabriel et al., 2018).

The JD–R model suggests that job demands will cause the loss of individual resources and undermine the effectiveness of workers’ own activities as they begin making mistakes and starting conflicts (Bakker, 2015). Red tape, as a job stressor, disrupts the efforts of community workers and diverts their resources and attention, often interrupting work goal progress. Meetings, filling out forms, and preparing for inspections interrupt the working rhythm of community workers and have a negative impact on the continuous progress of work, reducing their ability to devote themselves to their tasks (Rosenfeld, 1984). Some community workers complain that they have to manage all kinds of red tape at work, reducing the continuity of work and causing LOGP. Coping with the negative experience of red tape and adjusting to the interruptions of work require significant self-regulation resources, which are consumed at the expense of progress toward work goals. Therefore, we propose the following hypothesis.

*Hypothesis 2: Red tape has a positive impact on community workers’ LOGP.*

According to the JD–R model, jobs and behaviors that combine high demands with high resources are “active” jobs and behaviors. When job demands are high, job resources are particularly useful and motivating (Bakker and Demerouti, 2017). During the pandemic, community workers’ proactive behavior has had a high level of resource dependence. However, when community workers experience LOGP, they will devote less energy and resources to proactive behavior. Johnson et al. (2006) research shows that when individuals undergo cognitive transformations, the original process is often retained in their memories; it takes additional time and resources to revert to the previous working state after the work interruption, which makes it more difficult for individuals to maintain a positive attitude. In the prevention and control of COVID-19, community workers have often encountered red tape (Chen et al., 2021; Ezzat et al., 2021; Yuniza and Rebecca, 2021), which interrupts or delays the process of work and can limit community workers’ attention at a certain time (Kalisch and Aebersold, 2010). Instead of devoting energy, community workers tend to reduce active work and allocate the remaining resources to recent, specific, and time-limited daily activities. In light of this, we propose Hypothesis 3.

*Hypothesis 3: Community workers’ LOGP has a negative impact on their proactive behavior.*

Consistent with the above discussion, we further suggest that the relationship between red tape and proactive behavior will extend to the indirect relationship between red tape and proactive behavior through LOGP. A past empirical study shows that falling behind on work goals captures attention and spurs action aimed at reducing performance-goal discrepancies (Lord et al., 2010). When community workers perceive the lack of progress to be due to red tape, they will consider how and where they allocate their limited attentional resources (Neal et al., 2017). Thus, they have to balance the resources they deploy toward sustaining change-oriented behavior compared to more routine behavior. This may mean they will withdraw resources from proactive activities to accomplish other work goals. Therefore, we propose Hypothesis 4.

*Hypothesis 4: Community worker’s LOGP mediates the negative relationship between red tape and proactive behavior.*

### The Moderating Role of Public Service Motivation Between Lack of Goal Progress and Proactive Behavior

Public service motivation, a specific variant of prosocial motivation (Perry, 1996; Georgellis et al., 2011), has received a substantial amount of attention as both a predictor of individual and group performances. It is defined as an individual’s orientation to delivering services to others to improve societal welfare (Perry and Hondeghem, 2008). It is also a special form of intrinsic motivation and a stable individual personality trait (Perry and Vandenabeele, 2015). Since it is not “contingent on feelings of pleasure or enjoyment,” and instead, emphasizes the “meaning and purpose of the work” (Perry et al., 2010), scholars generally consider it a key psychological resource (Bakker, 2015) that can relieve work stress (Liu et al., 2015; Bao and Zhong, 2021). In public organizations, scholars believe PSM is the personal resource prerequisite for government employees’ engagement (Lavigna, 2015; Cooke et al., 2019).

Job demands–resources theory proposes that demands and resources (both job and personal) interact in predicting employees’ well-being and performance (Bakker and Demerouti, 2017). If workers face excessive job demands, strain and exhaustion increase, which may undermine performance unless they have enough resources available. If employees have sufficient job or personal resources, they are more engaged in their work, which facilitates performance and proactive behaviors, such as job crafting (Bakker, 2015). PSM plays an important role in offsetting burnout and work-related stress (Scott and Pandey, 2005; Mussagulova, 2021) and it weakens the links between work demands and performance. Those who with higher levels of PSM “will be better able to deal with organizational stressors because they know that dealing with those stressors serves the higher goal of helping others” (Bakker, 2015, p. 727). Therefore, in the management of the COVID-19 pandemic, community workers with higher PSM are not impervious to negative environmental conditions, but rather, they can deal with negative work situations more effectively (Hoek, 2021). They can adapt quickly after experiencing LOGP caused by red tape. They can seek opportunities to work on projects that have a significant impact on their community, even if the projects take up their time and energy (Rosen et al., 2019). On the basis of these arguments and literature review, we propose Hypothesis 5.

*Hypothesis 5: PSM moderates the relationship between LOGP and proactive behavior, such that the relationship is weak when community workers’ PSM is high.*

**FIGURE 1:**
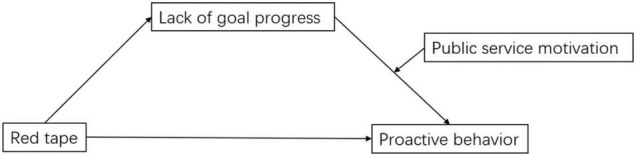
Theoretical model.

The theoretical framework is presented in Figure 1.

## Materials and Methods

### Participants and Procedures

The participants in this study consisted of full-time community workers in Beijing who are responsible for community pandemic prevention, alongside medical workers, volunteers, police officers, and other officials. They have been on the frontline of the fight against COVID-19 and their daily work has included community disinfection, dragnet screening of potential virus carriers, and helping households with difficulties. We contacted them through their participation in an online anti-pandemic training program. All the respondents indicated their willingness to participate by signing an informed consent form after we explained the purpose of the survey according to the Declaration of Helsinki. We distributed our online questionnaires through a Wechat group.

To avoid potential common method bias (Podsakoff et al., 2003), we collected data in two waves. Participants rated red tape and LOGP during the first wave. In the second wave, 2 weeks later, questionnaires on PSM and proactive behavior were distributed through the Wechat group. We used participants’ authorized nicknames in Wechat to match the responses from the two waves.

In total, excluding the questionnaires that could not be matched across the two waves, 656 participants provided complete responses, resulting in an effective response rate of 85.6%. Table 1 reports the demographic information for the sample. Among the employees sampled, 79.4% were women, 90.9% were married, and 42.4% had received a bachelor’s degree or above. The mean age was 42.42 years old (SD = 7.13), and the average work experience was 12.33 years (SD = 5.55).

**TABLE 1:** Basic demographic information of the sample (*N* = 656).

Gender	Male	20.58%
	Female	79.42%
Marriage	Unmarried	9.15%
	Married	90.85%
Education	High school and below	5.04%
	Junior college education	52.59%
	Undergraduate	41.46%
	Postgraduate	0.91%
Age (years)	Mean	42.42
	Median	43
	SD	7.13
	Min.	25
	Max.	63
Tenure (years)	Mean	12.33
	Median	12
	SD	5.55
	Min.	1
	Max.	38

### Measures

We used English-language scales and followed a strict translation and back-translation procedure. Each of these scales asked the community workers to rate their opinions on a 5-point Likert scale ranging from 1 (strongly disagree) to 5 (strongly agree).

#### Red Tape

We measured red tape using a validated 6-item scale translated into English by Borst et al. (2019). A high score indicates that an employee perceives a high level of red tape. A sample item is “Filling out forms and systems cost me a lot of time.”

#### Lack of Goal Progress

Lack of goal progress was measured with three items adapted from Koopman et al. (2016). A sample item is “Today, I have not made good progress on my work goals.”

#### Public Service Motivation

We adopted a five-item scale developed by Wright et al. (2013) to measure PSM. One of the items is “Meaningful public service is very important to me.”

#### Proactive Behavior

Community workers rated their proactive behavior using three items from Griffin et al. (2007). A sample item is “I come up with ideas to improve how my core tasks are done.”

#### Control Variables

Prior research has shown that demographic variables may influence LOGP (Neal et al., 2017) and proactive behavior (Smale et al., 2019). Thus, we controlled for gender, age, marriage, education, and tenure in our study.

## Results

### Data Analysis Strategy

We used the statistical software packages Mplus 7.4 and SPSS 25.0 to analyze the data. First, we conducted a confirmatory factor analysis (CFA) with Mplus 7.4. Then, we used hierarchical regression analyses to test the direct, mediating, and moderating effects in the model we specified. All descriptive, correlations and hierarchical regression analyses were analyzed using SPSS 25.0.

### Measurement Model

We conducted a series of CFAs (Muthen and Muthen, 2012) to ensure the satisfactory discriminant validity of red tape, LOGP, proactive behavior, and PSM. The results indicated that the hypothesized four-factor mode (χ^2^ = 100.768, df = 30, IFI = 0.983, TLI = 0.974, RMSEA = 0.06) was a better fit to the data than any other alternative models (see Table 2).

**TABLE 2:** Results of confirmatory factor analyses.

Model	χ^2^	df	χ^2^/df	△χ^2^	△df	RMSEA	TLI	IFI
1. Hypothesized four-factor model	100.768	30	3.359			0.06	0.974	0.983
2. Proactive behavior, PSM, and red tape-LOGP combined	193.504	33	5.864	92.736	3	0.086	0.946	0.961
3. PSM, red tape and LOGP-proactive behavior combined	541.596	32	16.925	440.828	2	0.156	0.824	0.875
4. Red tape, LOGP and PSM-proactive behavior combined	417.484	32	13.046	316.716	2	0.136	0.867	0.906
5. Red tape-LOGP and PSM-proactive behavior combined	505.594	34	14.870	404.826	4	0.146	0.847	0.884
6. Red tape and LOGP-PSM-proactive behavior combined	889.8	34	26.171	789.032	4	0.196	0.722	0.79
7. Red tape-LOGP-PSM combined and proactive behavior	481.162	34	14.152	380.394	4	0.142	0.855	0.89
8. Single-factor model	993.636	35	28.390	892.868	5	0.204	0.697	0.765

### Descriptive Statistics and Correlations

The means, standard deviations, correlations among variables, and reliability coefficients are shown in Table 3. Based on Cronbach’s alpha coefficients, all the scales exhibited internal consistency ranging from 0.82 for red tape to 0.94 for PSM, thus indicating acceptable internal consistency and reliability (α > 0.70). Among the control variables, marriage and education displayed positive correlations with red tape (*r* = 0.09, *p* < 0.05; *r* = 0.11, *p* < 0.01). This suggests that married and highly educated community workers perceived higher levels of red tape. As expected, red tape had a significant, positive correlation with LOGP (*r* = 0.65, *p* < 0.001) and a negative correlation with proactive behavior (*r* = −0.58, *p* < 0.001). LOGP was negatively related to proactive behavior (*r* = −0.58, *p* < 0.001). Moreover, PSM had a significant negative correlation with red tape (*r* = −0.24, *p* < 0.001) and LOGP (*r* = −0.21, *p* < 0.001) and a positive correlation with proactive behavior (*r* = 0.17, *p* < 0.001).

**TABLE 3:** Means, standard deviations, and correlations.

	Mean	SD	1	2	3	4	5	6	7	8	9
1. Gender	0.79	0.41									
2. Age	42.42	7.13	0.05								
3. Marriage	0.91	0.29	0.02	0.24***							
4. Education	2.38	0.6	–0.04	−0.36***	–0.07						
5. Tenure	12.31	5.54	0.14***	0.57**	0.16***	–0.08					
6. Red tape	3.88	0.7	0.02	–0.04	0.09*	0.11**	0.03	**0.82**			
7. LOGP	3.61	0.81	0.02	–0.01	0.06	0.04	0.01	0.65***	**0.87**		
8. Proactive behavior	2.57	0.92	0.05	0.06	–0.02	–0.06	0.03	−0.58***	−0.68***	**0.92**	
9. PSM	3.97	0.64	–0.03	–0.05	–0.03	0.04	0.01	−0.24***	−0.21***	0.17***	**0.84**

N = 656, *p < 0.05, **p < 0.01, ***p < 0.001. Reliability coefficients are shown in bold along the diagonal of the table.

### Hypotheses Testing

We conducted a hierarchical multiple regression analysis using SPSS 25.0 to test the hypotheses (Baron and Kenny, 1986). Table 4 shows the results of this analysis in detail.

**TABLE 4:** Results of hierarchical regression analyses.

	LOGP				Proactive behavior									
	β	SE	β	SE	β	SE	β	SE	β	SE	β	SE	β	SE
							
	Model 1		Model 2		Model 3		Model 4		Model 5		Model 6		Model 7	
Gender	0.02	0.08	0.01	0.06	0.04	0.09	0.06	0.07	0.06*	0.06	0.06*	0.06	0.06*	0.06
Age	–0.02	0.01	0.00	0.00	0.05	0.01	0.04	0.01	0.04	0.00	0.04	0.00	0.04	0.00
Marriage	0.06	0.11	0.00	0.09	–0.04	0.13	0.02	0.11	0.02	0.09	0.02	0.09	0.01	0.09
Education	0.03	0.06	–0.04	0.04	–0.03	0.07	0.03	0.05	0.01	0.05	0.01	0.05	0.01	0.05
Tenure	–0.03	0.00	–0.03	0.00	0.03	0.00	0.03	0.00	0.02	0.00	0.02	0.00	0.02	0.00
Red tape			0.66***	0.03			−0.58***	0.04	−0.23***	0.05	−0.23***	0.05	−0.23***	0.05
LOGP									−0.53***	0.04	−0.53***	0.04	−0.52***	0.04
PSM											0.01	0.04	0.01	0.04
LOGP*PSM													0.02	0.04
R^2^		0.01		0.43		0.01		0.34***		0.50***		0.50		0.50
△R^2^				0.42				0.33***		0.16***		0.00		0.00
F		0.77		81.85***		1.11		54.97***		90.68***		79.25***		70.49***

N = 656. *p < 0.05; ***p < 0.001.

Hypothesis 1 predicted that red tape was negatively related to proactive behavior. In Model 4, red tape was negatively and significantly related to proactive behavior (β = −0.58, *p* < 0.001), supporting Hypothesis 1.

Hypothesis 2 predicted that red tape was positively related to LOGP. Model 2 demonstrated that red tape was positively related to LOGP (β = 0.66, *p* < 0.001), which supports Hypothesis 2.

Model 5 showed that LOGP was negatively related to proactive behavior (β = −0.53, *p* < 0.001); this supports Hypothesis 3.

Hypothesis 4 predicted that LOGP would mediate the relationship between red tape and proactive behavior. Our analysis indicated that red tape was positively related to LOGP, and LOGP was negatively related to proactive behavior. Additionally, Model 5 showed that after adding the variable LOGP, the direct effect of red tape on proactive behavior was reduced (β = −0.23, *p* < 0.001). We further employed the PROCESS analysis and opted for Model 4 to test our mediating effect (Hayes, 2017). As shown in Table 5, the bootstrapping results showed that the indirect effect of red tape on proactive behavior through LOGP was significant (indirect effect = −0.453, 95% CI = [−0.415, −0.289]), thus providing support for Hypothesis 4.

**TABLE 5:** The mediating effect of lack of goal progress (LOGP) on the relationship between red tape and proactive behavior.

Effects	SE	Effect sizes	95% BC confidence LL	95% BC confidence UL
Total effect	–0.75	0.04	–0.84	–0.67
Direct effect	–0.30	0.05	–0.40	–0.21
Indirect effect	–0.45	0.04	–0.50	–0.37

*N = 656. Bootstrapping sample size = 5,000. SE, standard error; LL, bootstrapping lower limit confidence interval; UL, bootstrapping upper limit confidence interval.*

Hypothesis 5 predicted that red tape would moderate the relationship between LOGP and proactive behavior. Model 7 in our hierarchal multiple regression analysis showed that the interaction between LOGP and PSM had no significant correlation with proactive behavior (β = 0.02, ns); thus, Hypothesis 5 is not supported by the findings.

## Discussion

Since the outbreak of COVID-19, community-based management has been a key factor as an extension of governance for social management in restraining the spread of the virus. Operating at the grassroots level of a public organization, community workers have taken on arduous responsibilities and made important contributions. However, red tape severely hampers the initiative of community workers. To address this, the National Health Commission of the PRC issued a special notice on Effectively Reducing the Burden on the Grassroot Bureaucrats (National Health Commission, 2020). To examine how red tape impacts community workers’ proactive behavior, we applied the JD–R model to our study and explored the role of LOGP as a mediating variable and PSM’s moderating role. Empirical results from a two-wave investigation showed that red tape had a negative relationship with proactive behavior, and LOGP mediated the direct relationship. However, our hypothesis that PSM would moderate the relationship between LOGP and proactive behavior was not supported by the results. These findings have important theoretical and practical implications, which are discussed below.

### Theoretical and Practical Implications

Compared with previous studies, our study makes the following contributions. First, we answer recent calls to examine the practical usage of the JD–R model in public organizations (Lavigna, 2015; Perry and Vandenabeele, 2015). Scholars have conducted several studies based on JD–R theory, but they tend to focus on private rather than public organizations. As some scholars have pointed out, perceived red tape and PSM, as factors specific to the public sector, may affect civil servants’ job engagement and performance (Lavigna, 2015; Borst, 2018). Our study introduced these factors into the JD–R model as work requirements and personal resources to explore their influence on the behavior of community workers. Thus, our study enriches the JD–R model and broadens the underlying theory for explaining how community workers might behave given organizational demands and personal resources.

Second, using data from the unique period of the COVID-19 pandemic, we verified the negative impact of red tape on civil servants’ proactive behavior, which enriches prior research using two concepts. Although there is consensus among scholars that red tape has negative consequences (George et al., 2021), previous studies have mainly focused on employees’ job satisfaction, engagement, organizational commitment, and responsiveness (Torenvlied and Akkerman, 2012; Borst, 2018; Steijn and van der Voet, 2019). Our study took a step further toward resolving the negative effect of red tape on public employees’ proactivity in emergency circumstances, alerting scholars to examine other downstream outcomes that could be affected by red tape. Our study also broadens theory and research by assuming red tape as the negative antecedent variable. While, some studies have mentioned factors such as stressors at work (Ohly et al., 2006), ostracism (Williams, 2002; Ferris et al., 2008), and their significant influence on inhibiting employees’ proactive behavior, they tend to focus on leaders and individual workers. Our study focuses on institutional factors at the organizational level, revealing the negative impact of red tape on proactive behavior. This extends the literature on antecedent variables of proactive behavior. Interestingly, red tape does not just exist in public sectors; it exists in almost all kinds of bureaucratic organizations. Therefore, we encourage scholars to pay more attention to such variables that have previously received little attention.

Third, we examined the relationship between red tape and proactive behavior by identifying LOGP as one mechanism, which is different from other emotional and attitude variables, through which red tape operates. While previous studies have established the negative impact of red tape on individuals (George et al., 2021), the relationship between proactive behavior and red tape in the JD–R model, remains unexamined. According to our mediating framework, red tape as a job demand has a positive impact on LOGP because community workers must divert resources from other goal-relevant red tape tasks to check and filter. Dealing with these interruptions involves task switching, cognitive suppression, and emotion regulation, all of which consume resources (Lanaj et al., 2016). LOGP further has a negative effect on proactive behavior. Good accessibility and efficacy combined with goal progress are expected to produce beneficial well-being effects. When individuals perceive LOGP, they will strategically deploy their limited attention (Neal et al., 2017). Our results suggest that when community workers’ goal progress is impeded by red tape, they will withdraw energy and attention from behaviors that contribute to proactive behavior.

Finally, our results enrich the literature on PSM by showing that public employees with high levels of PSM do not necessarily show high levels of proactive behavior. In our study, the moderating effect of PSM between LOGP and proactive behavior was not significant. This seems odd because it contradicts the belief that PSM gives civil servants a greater sense of purpose, which helps them meet the demands of their jobs through organizing resources more efficiently, staying focused, and achieving job objectives (Bakker, 2015; Borst, 2018). A plausible explanation may be related to the dark side of PSM. Although most studies suggest that the effects of PSM are positive, its downside has been noted in research (Jensen et al., 2019). Empirical studies have demonstrated that PSM is not always positively associated with preferences for public sector employment (Hinna et al., 2021); public servants who exhibit higher levels of PSM may have higher turnover intentions when encountering work-family conflict (Hu et al., 2021). Consistent with Moynihan and Pandey (2007) conclusion that perceptions of red tape reduce PSM, we also found that red tape is negatively related to PSM. According to self-regulation theory, self-regulation is a process of controlling and regulating cognition, emotion, behavior, and psychology to achieve personal goals (Zimmerman, 2008). Individuals generally need to consume self-regulation resources to face various negative experiences. During the pandemic, community workers have become particularly averse to red tape; thus, when faced with a constant LOGP due to red tape, they exhaust their PSM as an individual resource and can no longer invest enough resources in proactive behavior. When confronted with obstacles inherent in their work environment, even workers with high PSM may not respond to these challenges in the same manner that they did prior to COVID-19 (Quratulain and Khan, 2015). In this case, since the moderating effect of PSM may not be significant, we encourage future research to further explore this issue.

Our study also has important practical significance if the mechanisms we identify are causal. First, as our results showed, red tape can significantly reduce community workers’ proactive behavior. In the fight against the COVID-19 pandemic, community workers’ routine and specific tasks provide a unique contribution, therefore, higher governments and leaders should be fully aware of the harm red tape can have on their proactive behavior and try to reduce unnecessary meetings, forms, and inspections. Second, as our paper highlighted, the LOGP mediates the negative relationship between red tape and proactive behavior. The government should recognize that proactive behavior has a high level of resource dependence and red tape constantly disrupts the normal routine of community workers, limiting the time and energy they can devote to learning and innovation and leaving them unable to cope with complex challenges in a rapidly changing environment. Finally, PSM does not mitigate the negative effects of red tape disruptions on proactive behavior. Therefore, instead of focusing on improving personal resources, governments should pay more attention to reducing red tape in formal institutions. China’s community workers are at the bottom of the bureaucratic hierarchy, and they must respond unconditionally and promptly to tasks assigned by higher authorities. So, when they are asked to attend meetings and fill out forms, they have no choice but to comply, regardless of whether the tasks are relevant to fighting the pandemic. Higher authorities in bureaucracy should re-examine existing working procedures and rules, assess their necessity and effectiveness, and simplify cumbersome and inefficient rules and procedures.

### Limitations

Despite its strengths, this study does have some limitations. First, we tested the relationship between red tape and proactive behavior; while based on Grant and Ashford (2008) definition, we did not differentiate between in-role or ex-role performance in proactive behavior. Correspondingly, we did distinguish between in-role goals and ex-role goals. This may have affected our judgment of the mediation mechanism of LOGP between red tape and proactive behavior. Therefore, future research should expand on our study by classifying different goals.

Second, contrary to our hypothesis, we found that PSM did not moderate the relationship between LOGP and proactive behavior. Although we explored the possible reasons for this, we did not further study the “black box” of the relationship. When Bakker and Demerouti (2017) reviewed the JD–R model studies, they pointed out that, besides having a direct positive effect on work engagement, personal resources are expected to buffer the undesirable impact of job demands on strain and boost the desirable impact of (challenge) job demands on motivation. The current study has provided only limited support for this proposition, which means that further research is needed to test the job demands and personal resources interaction.

Third, the data were only collected in Beijing, leaving it unclear whether our findings can be generalized to other cities or larger regions. Future research should expand sampling to include other locations.

Finally, the data were self-reported, having been obtained from questionnaires given to community workers. Future studies can use leaders’ evaluations of their employees’ proactive behavior as a data source to address the problem of homologous deviation.

## Data Availability Statement

The raw data supporting the conclusions of this article will be made available by the authors, without undue reservation.

## Ethics Statement

Ethical review and approval was not required for the study on human participants in accordance with the local legislation and institutional requirements. Written informed consent from the patients/participants was not required to participate in this study in accordance with the national legislation and the institutional requirements.

## Author Contributions

WH led the literature review, research design, and manuscript drafting work. SZ made contributions in literature and data analysis. SL made contributions in manuscript drafting work. All authors contributed to the article and approved the submitted version.

## Conflict of Interest

The authors declare that the research was conducted in the absence of any commercial or financial relationships that could be construed as a potential conflict of interest.

## Publisher’s Note

All claims expressed in this article are solely those of the authors and do not necessarily represent those of their affiliated organizations, or those of the publisher, the editors and the reviewers. Any product that may be evaluated in this article, or claim that may be made by its manufacturer, is not guaranteed or endorsed by the publisher.

## Appendix

### Survey Items

#### Red Tape

1. Filling out forms and systems costs me a lot of time.
2. It takes me a long time to comply with all the rules and obligations within my organization.
3. Some rules or guidelines that I encounter in my work contradict with each other.
4. Guidelines and regulations are more important in my organization than my experience or intuition.
5. Rules and procedures in my organization make it difficult to do my job well.
6. Requirements of supervisory bodies and inspections make it difficult for me to do my job well.

#### Lack of Goal Progress

1. I have made good progress on my work goals (R).
2. Things have not gone well with my work goals.
3. My work goals haven’t been going well.

#### Public Service Motivation

1. Meaningful public service is very important to me.
2. I am often reminded by daily events about how dependent we are on one another.
3. Making a difference in society means more to me than personal achievements.
4. I am prepared to make enormous sacrifices for the good of society.
5. I am not afraid to go to bat for the rights of others even if it means I will be ridiculed.

#### Proactive Behavior

1. Initiated better ways of doing my core tasks.
2. Come up with ideas to improve the way in which my core tasks are done.
3. Made changes to the way my core tasks are done.
